# Melanin Nanoparticles Obtained from Preformed Recombinant Melanin by *Bottom*-*Up* and *Top*-*Down* Approaches

**DOI:** 10.3390/polym15102381

**Published:** 2023-05-19

**Authors:** Sergio Alcalá-Alcalá, José Eduardo Casarrubias-Anacleto, Maximiliano Mondragón-Guillén, Carlos Alberto Tavira-Montalvan, Marcos Bonilla-Hernández, Diana Lizbeth Gómez-Galicia, Guillermo Gosset, Angélica Meneses-Acosta

**Affiliations:** 1 Laboratorio de Investigación en Tecnología Farmacéutica, Facultad de Farmacia, Universidad Autónoma del Estado de Morelos, Cuernavaca 62209, Morelos, Mexico; sergio.alcala@uaem.mx (S.A.-A.);; 2 Laboratorio de Biotecnología Farmacéutica, Facultad de Farmacia, Universidad Autónoma del Estado de Morelos, Cuernavaca 62209, Morelos, Mexico; 3 Farmacia Hospitalaria, Facultad de Farmacia, Universidad Autónoma del Estado de Morelos, Cuernavaca 62209, Morelos, Mexico; 4 Departamento de Ingeniería Celular y Biocatálisis, Instituto de Biotecnología, Universidad Nacional Autónoma de México, Cuernavaca 62209, Morelos, Mexico

**Keywords:** melanin nanoparticles, preformed recombinant melanin, nanocrystallization, high-pressure homogenization, double emulsion–solvent evaporation

## Abstract

Melanin is an insoluble, amorphous polymer that forms planar sheets that aggregate naturally to create colloidal particles with several biological functions. Based on this, here, a preformed recombinant melanin (PRM) was utilized as the polymeric raw material to generate recombinant melanin nanoparticles (RMNPs). These nanoparticles were prepared using *bottom-up* (nanocrystallization—NC, and double emulsion–solvent evaporation—DE) and *top-down* (high-pressure homogenization—HP) manufacturing approaches. The particle size, Z-potential, identity, stability, morphology, and solid-state properties were evaluated. RMNP biocompatibility was determined in human embryogenic kidney (HEK293) and human epidermal keratinocyte (HEKn) cell lines. RMNPs prepared by NC reached a particle size of 245.9 ± 31.5 nm and a Z-potential of −20.2 ± 1.56 mV; 253.1 ± 30.6 nm and −39.2 ± 0.56 mV compared to that obtained by DE, as well as RMNPs of 302.2 ± 69.9 nm and −38.6 ± 2.25 mV using HP. Spherical and solid nanostructures in the *bottom-up* approaches were observed; however, they were an irregular shape with a wide size distribution when the HP method was applied. Infrared (IR) spectra showed no changes in the chemical structure of the melanin after the manufacturing process but did exhibit an amorphous crystal rearrangement according to calorimetric and PXRD analysis. All RMNPs presented long stability in an aqueous suspension and resistance to being sterilized by wet steam and ultraviolet (UV) radiation. Finally, cytotoxicity assays showed that RMNPs are safe up to 100 μg/mL. These findings open new possibilities for obtaining melanin nanoparticles with potential applications in drug delivery, tissue engineering, diagnosis, and sun protection, among others.

## 1. Introduction

Melanin is an irregular, amorphous polymer of high molecular weight, generated by the oxidative polymerization of various indole and phenolic compounds such as L-tyrosine or levodopa (L-DOPA), which forms graphite-like planar sheets that aggregate naturally in a hierarchal fashion to form colloidal structures that can reach diameters of hundreds of nanometers [1]. The physical properties that make melanin unique are its light-absorbing ability, chemical resistance, electrical conductivity, and its low or null solubility in organic solvents or acidic aqueous media [1,2]. This natural polymeric pigment is distributed in all taxa of nature in compact granules integrated into the cytoplasm of different cells such as plant cell walls, some bacteria, fungi, the feathers of birds, and in the skin and hair of mammals [3]. Melanin has several biological functions, including protection against oxidizing agents [4,5], thermoregulation, the promotion of cell proliferation and differentiation [6]; it aids against osmotic and temperature changes [7], the capture of ultraviolet radiation [8], and it possesses chelating properties and immunomodulatory activities [9]. Several melanin types are derived from the evolutionary process: eumelanin is present in animals and humans as part of dark colors; pheomelanin is regularly associated with proteins to form chromoproteins that give a reddish-yellow coloration, and pyomelanin that is found in some fungi [10]. In humans, pheomelanin and pyomelanin are also found in other tissues and organs such as the retina, hair, brain, and liver [11]. Eumelanin is a brown-to-black pigment produced in humans by melanocytes (cells found in the basal layer of the epidermis in the skin) and it is distributed in granules ranging in size from 200 to 300 nm in the keratinocytes of the skin [12]. Knowledge of the melanin-formation route and of its properties as a polymer has enabled synthetic or biotechnological melanin to be obtained, which can be controlled in the physical characteristics or handled to generate new materials, such as copolymers, composites, or nanostructures [13,14].

Several studies have highlighted the potential use of melanin nanoparticles (MNPs) in different areas; their semiconductor properties have led to the generation of electronic films [15,16] and their high radiation-absorption capacity and antioxidant properties have been exploited to serve as adjuvants in cancer-radiation therapy [17,18] and sun protection against UV radiation [19,20]. In addition, they have also been suggested as drug carriers [21,22]. Other findings have shown an MNP affinity for metal ions such as Mg^2+^, Ca^2+^, and Na^1+^, acting as chelating agents, or with the ability to trap metal ions with a potential toxic effect, such as Cd^2+^ and Pb^2+^ [23]. Furthermore, MNPs present good photoacoustic properties that have been used for the diagnosis of breast tumors [24,25] and as a contrast agent in optoacoustic tomography [26]. Other studies have demonstrated that MNPs could protect hematopoietic cells from free radicals induced by gamma radiation in mice [27]. Additionally, along with developing new biomaterials, such as biodegradable polymers, MNPs have become useful in tissue engineering, serving as a support and guide for proper cell growth by forming biofunctional structures [28].

The most common manufacturing process for obtaining synthetic MNPs reported in the literature has mainly been the *in-situ* polymerization method by the oxidation of L-tyrosine, its natural precursor. However, biotechnology and genetic engineering have made it possible to obtain melanin in industrial quantities from recombinant sources, with the advantage of knowing its chemical, physical, and physicochemical properties prior to material manipulation in a nanoparticle manufacturing process [29,30], favoring homogeneous production and improved quality control. This preformed raw material can be controlled in molecular weight and chemical structure, and due to its mechanical and thermal resistance, may be nanosized using different techniques without the necessity of polymerization reactions, allowing its use in large quantities.

On the other hand, there are two different approaches to address the nanotechnology, that is, *bottom-up* and *top-down*, which include all the existing manufacturing methods to generate nanoparticles. For the selection of the best approach, it is necessary to know the raw material in terms of the solid state, chemical behavior, solubility, pH dependence, and mechanical or thermal behavior [31]. The *bottom-up* approach starts from the molecular or atomic state of matter, employing methods that benefit the assembly or aggregation of material until the nanostructure is achieved. A common feature in the methods developed by this approach is that precursors or raw materials should be in molecular dispersion, that is, previously dissolved, to permit the addition of an antisolvent, or create an environment where nanoparticle formation is promoted; the use of surfactants or stabilizers plays an essential role in these methods [32]. Technologies such as microemulsion, single or multi-emulsion (double)—with subsequent evaporation, extraction, or diffusion solvent, as well as interfacial emulsion polymerization and nanoprecipitation—are included in this approach [33,34]. The *top-down* approach refers to the production of nanostructures from macrometric materials by means of size reduction through various techniques such as milling media, fragmentation, cutting or engraving, machining film processes, photolithography, printing, or homogenization at high or ultra-pressure. These manufacturing methods require large amounts of energy and generate large amounts of heat, posing a great danger to labile materials [31,32].

In this work, recombinant melanin nanoparticles (RMNPs) were obtained utilizing three different manufacturing methods (nanocrystallization, double emulsion–solvent evaporation, and high-pressure homogenization), by manipulating a preformed recombinant melanin (PRM) as a new raw polymeric material, with the aim of evaluating and exploring its capacity to be utilized to generate nanoparticles when these manufacturing approaches are applied, as well as of evaluating the effect of the process parameters on the physical properties of the nanoparticles. Methods were optimized to generate RMNPs with a target size of ~250 nm using statistical techniques. The nanoparticles were characterized by their physical, chemical, physicochemical, and in vitro biocompatibility properties.

## 2. Materials and Methods

### 2.1. Materials and Cell Lines

Preformed recombinant melanin was donated by the Institute of Biotechnology—UNAM. Briefly, melanin was obtained using a platform of recombinant *Escherichia coli*, expressing the tyrosinase gene from *Rhizobium etli* by Chávez-Béjar et al. [35]. Sodium hydroxide, ammonium hydroxide and hydrochloric acid were purchased from JT Baker (Monterrey, Mexico) (>99% pure; analytical reagent). Solvents such as propanol, hexane, benzene, dichloromethane, glyceryl triacetate, n-butanol, octanol, ethyl acetate, ethanol, acetone, isopropyl alcohol, and isobutyl alcohol were supplied by Fermont (Monterrey, Mexico) and Sigma-Aldrich (Saint Louis, MO, USA) (>98% pure; reagent grade). Polyvinyl alcohol (PVAL, Mowiol 4-88) and polysorbate 80 (Tween 80), used as stabilizing agents, were supplied by Sigma-Aldrich (Saint Louis, MO, USA). Distilled water from a Milli-Q filtration system was used throughout the experiments (Millipore, Billerica, MA, USA).

Two types of certified cell lines were used: human embryonic kidney (HEK293) (ATCC CRL-1573) and human epidermal keratinocytes from normal neonatal foreskin (HEKn) (ATCC PCS-200-010). HEK-293 cells were cultivated using Dulbecco’s Modified Eagle’s Medium/Nutrient Mixture F-12 (DMEM/F12-GIBCO) supplemented with 10% FBS (GIBCO), 3.574 g/L of (4-(2-hydroxyethyl)-1-piperazinaetanosulfonic [HEPES]), and 2.44 g/L of sodium bicarbonate. HEKn cells were grown using Dermal Cell Basal Medium (DCBM) (ATCC PCS-200-030) supplemented with a specific Keratinocyte Growth Kit (ATCC PCS-200-040) in T-75 culture flasks in an atmosphere of 5% CO_2_ at 37 °C, according to the instructions provided by the manufacturers. Percentage of cell viability was determined using two different standard methods such as Trypan Blue staining (Sigma-Aldrich; Saint Louis, MO, USA) and MTS (CellTiter 96® Aqueous One Solution Cell Proliferation Assay; PROMEGA, Madison, WI, USA).

### 2.2. Characterization of Preformed Recombinant Melanin (PRM) 

PRM was previously generated by Chávez-Béjar et al. [35,36], through a bioprocess in which plasmid *pTrcMutmelA* with the *MutmelA* gene of *Rhizobium etli* that encodes for the enzyme tyrosinase was used to transform *Escherichia coli W3110* into *E. coli W3110*/*pTrcMutmelA*, a strain capable of producing eumelanin from L-tyrosine as substrate. The donated melanin was a dark brown-colored material, with an irregular shape and an amorphous granular aspect (Figure S1A). One hundred g of this preformed melanin was placed in a mortar and pulverized for 10 min until a fine powder was obtained (Figure S1B). This powder was used as the raw material in all further studies and characterized in terms of chemical identity, apparent solubility, and capacity to be sterilized by wet steam to define the process conditions in the melanin-nanoparticle manufacturing methods.

#### 2.2.1. Melanin Identity by FTIR and UV–Vis Spectrophotometry

The IR and UV–Vis spectra of the PRM were obtained. In brief, for IR studies, 2 mg of pulverized PRM was placed onto the ATR implement of the FTIR spectrophotometer (ABB-MB3000 FTIR, ABB Group, QC, Canada). Then, 150 scans per sample were measured, in the range of a wavenumber of 500–4000 cm^−1^, with a resolution of 16. The reported IR spectrum represents the average of the scans. To record the UV–Vis spectrum, a PRM solution was prepared from 25 µg/mL in 1 N NaOH, with the scanning of the light absorption in the UV–Visible region of the electromagnetic spectrum, in a wavelength range of 200–800 nm, utilizing a double-beam Cary 60 UV–Vis spectrophotometer (Agilent, Santa Clara, CA, USA), employing 1 N NaOH solution as blank. The maximal absorption wavelength for PRM was set from the UV spectrum. PRM identity was verified by comparing the obtained spectra with the melanin IR and UV spectra previously reported in the literature.

#### 2.2.2. Apparent Solubility

The apparent solubility of PRM in different solvents was carried out to determine the subsequent approach and strategy to generate recombinant melanin nanoparticles (RMNP). Briefly, 10 mg of PRM raw material was added to a series of 10 mL test tubes, which were labeled with the test solvent. Then, increasing amounts of 100 µL of different dissolution media, organic solvents (of different polarities) or aqueous media (at different pHs), were added until complete dissolution was observed, or a maximal volume of 10 mL was reached. All samples were shaken vigorously with the aid of a vortex. When 10 mL of solvent was used and no dissolution was observed, the result was taken as “practically insoluble in the medium”. Tests were performed in triplicate, reporting an average in mg/mL.

#### 2.2.3. Autoclavable Resistance

To assess the resistance of the PRM raw material to the wet steam sterilization process, three samples of 2 g of powder, placed in a 10 mL test tube, were sterilized in a Hinotek autoclave (YXQ-LS-SII Vertical type; Hinotek, Ningbo, China), for 20 min at 121 °C and at 15 psi of pressure. Afterward, to evaluate the chemical stability, the IR spectra of the samples were obtained, employing the methodology mentioned previously.

### 2.3. Obtaining Recombinant Melanin Nanoparticles (RMNPs) by a Bottom-Up Approach

Since the manufacture of nanoparticles under this approach requires manipulation of the material at a molecular level, the results of apparent solubility were considered to select the manufacturing methods. Due to melanin being only soluble in alkaline aqueous media (pH > 12), the manufacturing methods of nanocrystallization and double emulsion–solvent evaporation were selected, where the dissolution medium or the aqueous phase was a 1N of NaOH solution.

#### 2.3.1. Nanocrystallization Method (NC)

The preparation of RMNPs by the nanocrystallization method (nanoprecipitation) was performed using an adaptation of the methodology proposed by Chen et al. [31,37]. Nanoprecipitation is based on generating a non-solvent environment for the material that is to be nanosized. Here, an alkaline PRM solution (at a concentration of 1 mg/mL in 1N NaOH) was neutralized with an acidic solution (HCl) of PVAL (stabilizing agent), utilizing a dosing unit (Dosimat 665; Metrohm AG, Herisau, Switzerland). The PRM alkaline solution was poured into the acid solution, at a rate of 14 µL/s and constant stirring at 600 rpm. To evaluate the effect of the manufacturing-process parameters on the characteristics of the RMNPs, a 2^K^ experimental design (k = 3) was established with the following factors and levels: X_1_: volume of the acidic aqueous phase (10 mL or 30 mL); X_2_: concentration of HCl (0.5 N or 1 N), and X_3_: the concentration of stabilizing agent (PVAL at 1% or 5% *w*/*v*). Table 1 presents the design matrix and the total experimental runs. The volume of the alkaline PRM solution (10 mL) and the stirring time after neutralization (2 h) (Dual-Range Mixer IKA RW20 digital, IKA-Werke, Satufen, Germany) remained constant in all experiments. The formed RMNPs were obtained in aqueous suspension; hence, the nanoparticles were washed three times by centrifugation at 3000× *g*, 4 °C, and 60 min (Eppendorf 5804R centrifuge, 15-amp version, Thermo Fisher Scientific, Waltham, MA, USA), then the nanoparticles were resuspended in each wash with 5 mL of distilled water. Average particle size (Y_1_) and Z-potential (Y_2_) were measured as response variables in all runs.

#### 2.3.2. Double Emulsion–Solvent Evaporation Method (DE)

Due to the nonsolubility of melanin in organic solvents, the double emulsion–solvent evaporation method proposed by Liu et al. [38] was adapted to produce the RMNPs. Briefly, a W1/O first emulsion was prepared by homogenization at high shear, whereby the first aqueous phase (W1), composed of an alkaline solution of PRM (1 mg/mL, in 1 N NaOH) and a stabilizing agent (Tween 80), was dispersed into an organic phase (O) composed of dichloromethane or a mixture of dichloromethane acetone. This emulsion was then homogenized under gentle stirring in a second aqueous phase (W_2_), comprising an acidic solution of HCl and a second stabilizing agent (PVAL). A neutralized W_1_/O/W_2_ emulsion type was formed. Evaporation of the O phase in conjunction with the phenomenon of neutralization caused the generation of the RMNPs as an aqueous suspension. 

The manufacture of RMNPs by this method was optimized following a 2^k^ experimental design (k = 3), which was established with the following factors and levels: X_1_: volume of O phase (mix of dichloromethane [DCM]-acetone [ACE]) (10 mL or 20 mL); X_2_: DCM-ACE ratio in the O phase (at proportions of 1:0 or 2:1), and X_3_: the homogenization time in the first emulsion (5 min or 10 min). Table 1 depicts the design matrix and the experimental runs. The first W_1_/O emulsion was generated using an UltraTurrax homogenizer (IKA T18 Digital, IKA-Werken, Staufen, Germany) at 12,000 rpm according to the predefined homogenization times in the experimental design. Then, the first emulsion was poured into 40 mL of an acidic PVAL solution (HCl at 0.25 N), stirring at 600 rpm (Dual-Range Mixer IKA RW20 digital, IKA-Werken, Staufen, Germany) for 30 min. At the end of the stirring time of the second emulsion, the O phase was evaporated entirely under reduced pressure, using a rotary evaporator (IKA RV 10) at 90 rpm and 30 °C. During the experiments, the concentration of the stabilizing agents in the first and the second emulsions (Tween 80 at 5% *v*/*v*, and PVAL at 1% *w*/*v*, respectively) and the volume of the aqueous phase W1 (10 mL) in the first emulsion remained constant. In each experimental run, the RMNPs were recovered by centrifugation at 3000× *g*, 4 °C, and 60 min (Eppendorf 5804R centrifuge, 15-amp version) and washed three times to eliminate stabilizing agent residues, resuspending each wash with 5 mL of distilled water. Average particle size (Y_1_) and Z-potential (Y_2_) were measured as response variables in all runs.

### 2.4. Obtaining Recombinant Melanin Nanoparticles (RMNPs) by a Top-Down Approach

To produce RMNPs using this approach, the material should be reduced to nanoscale size by milling, collision, attrition, and sometimes cavitation. Considering the crystalline structure of PRM and the possibility of reducing its size by pulverization with a mortar, RMNPs were manufactured with the following technique:

#### High-Pressure Homogenization Method (HP)

A high-pressure homogenizer (Microfluidizer LM 10, Microfluidics International Corporation, Newton, MA, USA) was used. Particle size reduction was evaluated through a 2^k^ experimental design (k = 2), with one central point. Factors and levels considered in the study included X_1_: applied pressure (12,000 or 23,000 psi), and X_2_: number of cycles (5 cycles in the first step or 10 cycles in the final stage). One cycle corresponds to the complete passage of the samples from the hopper through the equipment’s impact micro-camera until its exit. A volume of 40 mL of an aqueous suspension of PRM was treated at the predefined pressure and cycles. The average particle size (Y_1_) and Z-potential (Y_2_) were immediately measured in the final aqueous suspension. Design matrix and experimental runs are included in Table 1.

### 2.5. RMNP Optimization and Recovery

Once the RMNPs were obtained by the three evaluated technologies, the optimal manufacturing conditions were defined by response surface methodology (RSM) and desirability function for multiple responses. The combination of levels was estimated for the factors through which RMNPs with an average particle size of 250 ± 50 nm and high Z-potential were reached; these values were fixed as optimization objectives. The average particle-size target was established to mimic the nanometric natural size of melanin granules in keratinocytes, and a high Z-potential was desired to assure good stability in suspension (expecting ± 30 mV). The predicted levels for each method are presented in Table 2. Three batches of RMNPs were manufactured under the optimized conditions to verify the predictive model: the prepared nanoparticles were identified as RMNP-NC, RMNP-DE, and RMNP-HP. 

As the final step in the manufacturing process, the RMNP suspensions were washed three times with 5 mL of distilled water, using centrifugation cycles (at 4 °C, 15,557× *g*, and 60 min). To prevent particle aggregation after centrifugation, all RMNP samples were sonicated using an ultrasonic probe (SONICS Vibra Cell VCX 130, Sonics and Materials Inc, Newtown, CT, USA) at 10 kHz, for 30 min. Finally, these aqueous suspensions of RMNPs were lyophilized. Briefly, all samples were frozen for 3 h on dry ice, and placed on a bulk rack in the condenser at −80 °C in the lyophilizer (BenchTop Pro with Omnitronics, SP Scientific VirTis, Warminster, PA, USA). Vacuum was applied to reach 200 mTorr for 48 h. These dried nanoparticles, that is, RMNP-NC, RMNP-DE, and RMNP-HP, were employed to conduct subsequent physical characterization studies and biocompatibility assays.

### 2.6. Physical Characterization of RMNP

#### 2.6.1. Particle-Size Analysis

The average particle size of the RMNPs and their distribution, for all experiments, was determined utilizing the dynamic light scattering technique (DLS) of Zetasizer equipment (Nano ZS90, Malvern Panalytical, Malvern Worcestershire, UK). Operational conditions included were distilled water as dispersing medium (refractive index of 1.33), with an equilibration time of 120 s, a diffraction angle of 90° at 25 °C, with a resolution of 16 readings, and in triplicate. Samples were diluted at 1:10 using the same dispersing medium, placing 1 mL of suspension in polystyrene cuvettes.

#### 2.6.2. Zeta-Potential Measurement

Zeta-potential measurement was performed using the electrophoretic light scattering technique under similar conditions to those of the average particle size, using capillary polystyrene cuvettes (Zetasizer Nano ZS90; Malvern Panalytical, Malvern Worcestershire, UK). Measurements were performed on samples that were diluted at a 1:5 ratio using distilled water as dispersing medium.

#### 2.6.3. Morphological Analysis

The morphology of RMNPs was described using transmission electron microscopy (TEM). A volume of 25 µL of each RMNP suspension was placed on a copper grill and was dried at room temperature, adding uranyl acetate as a contrast solution. The micrographs were recorded with an electron microscope (Zeiss EM900 80 kV, Carl Zeiss AG, Oberkochen, Germany) in conjunction with a Dual Vision 300 W CCD camera (Gatan, Inc, Warrendale, PA, USA).

#### 2.6.4. Evaluation of Solid State

This characterization was carried out using the lyophilized RMNPs (RMNP-NC, RMNP-DE, and RMNP-HP).

##### ATR-FTIR Studies

A mass of approximately 2 mg of dried nanoparticles was placed on a horizontal ATR SeZn attachment in the FTIR spectrophotometer (ABB-MB3000 FTIR; ABB Group, QC, Canada). IR spectra were obtained in the vibrational wavenumber range from 500 to 4000 cm^−1^, with a resolution of 16 and 150 scans.

##### Powder X-ray Diffraction (PXRD) Analysis

PXRD analyses were performed for PRM and for all of the prepared RMNPs in the transmission mode on a Bruker D-8 Advance diffractometer (Bruker, Billerica, MA, USA) equipped with a LynxEye detector (λCu-Kα1 = 1.5406 Å, monochromator: germanium) (Bruker, Billerica, MA, USA). The equipment was operated at 40 kV and 40 mA, and data were collected at room temperature in the range of 2θ = 5–40°. 

##### Calorimetric Studies by Differential Scanning Calorimetry (DSC)

Thermal analysis was performed on PRM raw material and dried RMNPs using the DSC technique, with a Q20 Calorimeter (TA Instruments, New Castle, DE, USA). Briefly, a mass of 5–10 mg of each sample was placed in a Tzero aluminum hermetic pan (TA Instruments, New Castle, DE, USA) that was sealed with a press machine. Experimental assays were carried out under the following operating conditions: thermal equilibrium at 15 °C; temperature range from 20 to 300 °C; a heating rate of 10 °C/min, and an inert environment with nitrogen at a flow of 50 mL/min.

### 2.7. Biocompatibility Evaluation: In Vitro Cytotoxic Assay

Based on physical characterization, RMNP-DE were chosen as ideal for cytotoxic evaluation due to their morphology and reproducibility. Evaluations were made on HEK293 and HEKn cell lines. MTS methodology was used to measure cell activity, and cell viability in the presence of RMNP, and the cell counting was performed with the Trypan Blue technique. Briefly, 20,000 cells per well were seeded in 96-well plates, using DMEM/F12 medium supplemented with 10% FBS at 37 °C and 5% CO_2_, which were incubated to promote cell adherence. After 24 h of incubation, different RMNP-DE concentrations (10, 50, and 100 µg/mL) were added to different wells and were then incubated for an additional 72 h; a well with no treatment was set as control. Lastly, the culture medium was eliminated carefully, and 90 µL of DMEM/F12 and 10 µL of MTS were added and incubated for 4 h. The absorbance of the supernatant was measured at 490 nm utilizing an Epoch microplate spectrophotometer (Biotek, Agilent Technologies, Santa Clara, CA, USA). 

### 2.8. Statistical Analysis

Multiple regression analyses were performed using response surface methodology, with an analysis of variance (ANOVA) to assess significant effects and model fit. One-way ANOVA was also employed to compare groups, with the Dunnett post hoc test. Statistically significant differences were considered when *p* < 0.05. All statistical analyses were performed using Statgraphics statistical software (Version Centurion XVIII).

## 3. Results and Discussion

### 3.1. PRM Characterization

The recombinant melanin raw material produced in *E. coli W3110/pTrcmelA* was characterized by identity, autoclavable resistance and apparent solubility, because it is a material with a different origin from that of conventional melanin. PRM has a macroscopic physical appearance as a dark-colored irregular and granular material that, after being pulverized, produced a resulting particle size of 7.5 ± 2.9 µm (Figure S1). This initial grinding made it possible to obtain a homogeneous raw material, with an increased surface area, and one that enhanced the dissolution rate during the apparent solubility test.

**Figure 1:**
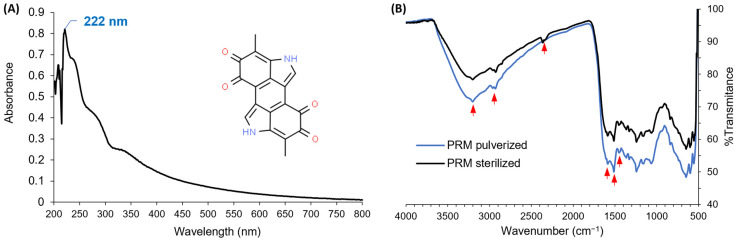
Spectra of PRM: (**A**) UV–Vis spectrum, (**B**) IR spectrum; the red arrows show the absorption peaks related to the known functional groups of melanin.

The UV–Vis and IR spectra of PRM are revealed in Figure 1. The UV–Vis spectrum of PRM previously dissolved in an alkaline solution evidenced that the maximal UV-light absorbance occurs at a wavelength of 222 nm (Figure 1A), which is in agreement with the reports of Tan et al. [39], Dong and Yao [40], and Madhusudhan et al. [41]. Regarding the FTIR studies, the IR spectrum of the PRM demonstrated absorption peaks related to the known functional groups of melanin (Figure 1B); a signal at 3200 cm^−1^ is related to a broad band of the -NH and -OH groups stretching; peaks at 2920 cm^−1^ and 1450 cm^−1^ correspond to the -CH stretching and bending vibration, respectively; a strong signal attributed to the -C=O bond in -COOH groups was observed at 1595 cm^−1^; vibration of the -C=C- bonds in the aromatic groups is associated with a peak at 1500 cm^−1^; and finally, a peak at 2385 cm^−1^ can be recognized as the protonated amine of 5,6-dihydroxyindole-2-carboxylic acid (characteristic monomer in melanin). The peaks of these spectra correspond to those reported in the literature [39,40,41,42,43]. These findings allowed the identity of the melanin to be verified as eumelanin. In addition, as depicted in Figure 1B, PRM after the wet-steam sterilization process does not present any differences in its chemical structure, in that no changes in the IR spectrum were observed. This sterilization resistance is relevant when biocompatibility studies are executed or if biomedical applications are considered for this material.

The results of PRM solubility in different solvents are summarized in Table 3. The insoluble nature of melanin was observed in a wide range of solvents with different polarities, except for alkaline aqueous media (high pH, >12, either 1N NaOH or 1N NH_4_OH); however, the results classify it as slightly soluble according to United States Pharmacopeia (USP) criteria. Ye et al. and Guo et al. also reported this solubility behavior for natural and conventional melanin [44,45], which were materials obtained from a source other than a recombinant origin.

The determination of the apparent solubility of PRM was an essential aspect because it provided a starting point for designing a manufacturing strategy to obtain recombinant melanin nanoparticles (RMNPs). This was particularly apparent in the *bottom-up* approach, where materials must be manipulated at a molecular level. Based on these solubility results, two methods widely utilized in the development of nanocrystals were adapted: nanoprecipitation and emulsification–solvent evaporation. For the latter, a double emulsion alternative was chosen, because the nanostructure shape and size could be controlled from the first emulsion, it being possible to neutralize this first aqueous phase in a second emulsion because the organic solvent is evaporated, creating an anti-solvent effect for the PRM, which leads to the formation of the nanoparticles.

On the other hand, the aqueous-insoluble character of the PRM also facilitated the selection of the dispersing medium to generate nanoparticles in the *top-down* approach. For this project, it was desirable to achieve an aqueous suspension of nanoparticles; thus, distilled water was chosen as the vehicle in the manufacture of RMNPs by the high-pressure homogenization process.

### 3.2. Melanin Nanoparticles by Bottom-Up and Top-Down Approaches

Three nanoparticle manufacturing methods, including nanocrystallization (NC), double emulsion–solvent evaporation (DE), and high-pressure homogenization (HP), were adapted to prepare nanomaterials from preformed recombinant melanin (PRM). Table 1 presents the design matrix generated and executed for each method; average particle size and Z-potential are reported for all experimental runs. 

For the manufacturing processes with a *bottom-up* approach, certain similarities could be observed in the behavior of the average particle size. The NC method generated RMNPs with a size ranging from 160.0 to 958.6 nm, and with Z-potential between −1 and −30 mV. Meanwhile, with the DE method, the RMNPs were obtained with a size distribution range of 167.5–874.3 nm but, unlike the previous method, a more homogeneous Z-potential was observed in the different runs, with values between −18 and −40 mV, which were ideal for good physical stability for the nanoparticles in aqueous suspension. For both methods, experimental run #4 produced RMNPs sufficiently close to the expected target size of 250 ± 50 nm (Table 1). 

On the other hand, the top-down approach was applied to reduce the particle size of the pulverized PRM by the application of the HP method to prepare melanin nanoparticles (RMNPs). As shown in Table 1, this technique led to average particle sizes ranging from 317.2 to 505.1 nm, with homogeneous Z-potential values of between −35.3 and −41.6 mV. However, it was not possible to produce nanostructures smaller than 300 nm, even though operating conditions at the highest working pressure and the greatest number of cycles in the high-pressure homogenizer were utilized. For this reason, the manufacture of the optimized RMNPs employing the HP method was fixed at operational conditions under which the smallest particle size was generated, meaning experimental run #1.

**Figure 2:**
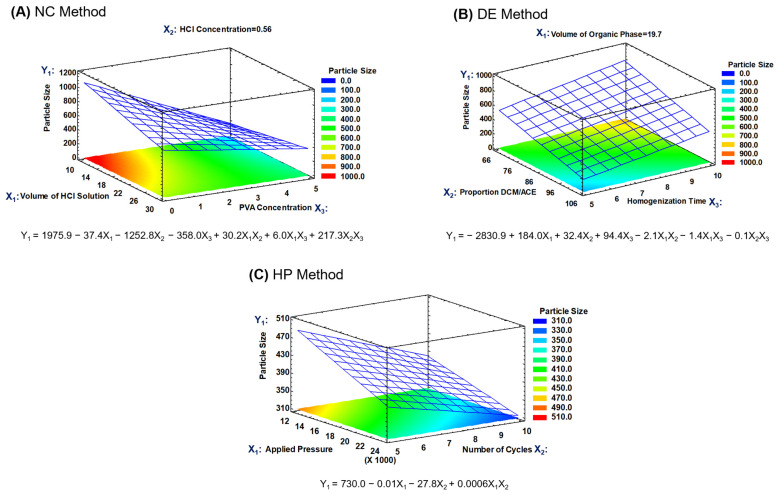
Response surfaces for the particle size of the recombinant melanin nanoparticles (RMNPs) for the three applied manufacturing methods. (**A**) Nanocrystallization (NC), (**B**) double emulsion–solvent evaporation (DE), and (**C**) high-pressure homogenization (HP).

Regarding the statistical analysis of the main effects of the factors, this was focused on particle size, in that the main objective was to produce nanoparticles of a specific size (Figure S2). However, during the optimization process, both variables were considered (particle size and Z-potential), carrying out predictions with the fitted models, searching the levels for the studied factors in which the target particle size and a maximal Z-potential could be reached. High Z-potential favors the physical stability of RMNPs in aqueous suspension. Hence, response surface methodology was utilized to define the most appropriate regression model. The response surfaces for the particle size of the RMNPs and the fitted mathematical models for all of the applied methods are presented in Figure 2.

To prepare RMNPs, the stabilizing agent concentration (X_3_) was the factor that mainly affected the particle size in the NC method, generating a decrease in the average size as the PVAL concentration increased. This effect is due to the stabilizing effect of the PVAL on the solid–liquid interface during the precipitation process, in that its steric stabilization mechanism exerts an influence on the crystal growth during the formation of the nanoparticle [46,47]. Regarding the neutralization process, it is suggested that chemical equilibrium in some cases was reached, and in others, there was a change in pH sufficiently close to neutrality that it created an anti-solvent environment for melanin, leading to the precipitation of the dissolved material [31]. The volume of the acid medium and the concentration of the neutralizing agent, factors X_1_ and X_2_, respectively, were not significant, but a slight tendency was observed toward the generation of a larger average size when the volume of the acidic medium was increased. Thus, based on these findings, the response surface for the particle size was obtained with the factors X_1_ and X_3_ (Figure 2A), with the factor X_2_ remaining at a low level; as can be observed, a second-order model was fitted with a determination coefficient (R^2^) of 0.944.

Following the same strategy of analysis, during the preparation of RMNPs by the DE method, a significant effect on the particle size was observed when the homogenization time (factor X_3_) was increased in the first emulsion. Because homogenization of the phases is conducted in a high-shear process, the reduction in the droplet size of the discontinuous W_1_ phase (aqueous alkaline solution that contained the previously dissolved PRM) revealed time dependence; the latter is relevant because the droplet size in the first emulsion plays an essential role in reaching a desired particle size [48,49]. Regarding factors X_1_ and X_2_, the volume of the O phase and its composition (DCM:ACE proportion), respectively, did not exert a significant impact on the particle size. Moreover, the neutralization of aqueous phase W_1_ with the W_2_ acidic aqueous phase occurred as the O phase was evaporated from the W_1_/O/W_2_ emulsified system, generating an anti-solvent environment for the previously dissolved RPM, leading to the formation of the RMNPs. Figure 2B depicts the response surface for particle size for the RMNPs produced by the DE method, where factors X_2_ and X_3_ were employed to construct the graph and explore the surface, with factor X_1_ remaining at a low level, in that its effect was practically null; a second-order model with a R^2^ of 0.946 was found in a similar manner.

The experimental design of two factors was executed to produce RMNPs by a top-down approach which evidenced non-statistically significant differences in particle size at the pressure and the number of cycles set in the high-pressure homogenization process. Nevertheless, a tendency was detected for the particle size to be reduced as the applied pressure (factor X_1_) or the number of cycles (factor X_2_) increased. Additionally, a common reduction limit was observed in the particle size, independent of the high pressure or the number of times that the PRM suspension passed through the impact chamber in the equipment [50]. The response surface for the particle size when the HP method was applied is presented in Figure 2C; a second-order model exhibited the best fit, with an R^2^ of 0.647. Thus, based on these findings and on the operational conditions tested in this manufacturing method, the RMNPs obtained under these experimental conditions provided the smallest particle size observed (run #1).

### 3.3. Optimization of RMNP Particle Size and Model Verification

Using the fitted regression model of each previously analyzed surface, the optimization process was carried out for each manufacturing method applied to produce RMNPs. From the DOE analysis menu in the Statgraphics software, the response optimization tool was used to make predictions for the levels of the factors in which it was possible to achieve the established optimization objectives, that is, a particle size of 250 nm and a Z-potential with the highest value possible. Optimization by multiple responses was performed using the desirability function, giving the greater weight for the particle size in the equation, mainly to achieve this objective. Once the optimized levels for each factor in the models were theoretically estimated, the expected or predicted particle size was determined for the estimated conditions. To verify that the models adequately predicted the behavior of the particle size, three batches of RMNPs were prepared under the optimized conditions for each manufacturing method, measuring the response variables, and comparing the predicted values versus the experimental values. The prediction error was determined by the difference in values between the expected and the experimental responses. The results of the estimates, optimization conditions, desirability, and model verification are summarized in Table 2. The RMNPs obtained under the estimated conditions were recognized as the optimized nanoparticles.

### 3.4. Physical Characterization of the Optimized RMNPs

**Figure 3:**
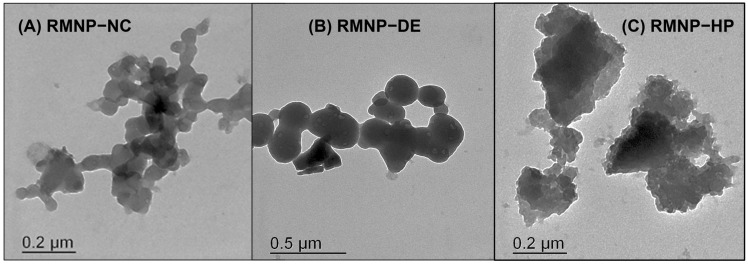
TEM micrographs of the recombinant melanin nanoparticles (RMNPs) obtained by the following methods: (**A**) NC = nanocrystallization; (**B**) DE = double emulsion–solvent evaporation, and (**C**) HP = high-pressure homogenization.

Morphological studies to verify the particle size and to determine the shape of the RMNPs were carried out using the transmission electron microscopy (TEM) technique. The obtained micrographs are presented in Figure 3. It can be observed how the RMNPs produced under the *bottom-up* approach (Methods: NC and DE) exhibited a similar structure. These were nanoparticles with a spherical, homogeneous, and solid shape (Figure 3A: RMNP-NC, and 3B: RMNP-DE). It is worth mentioning that the particle size observed in TEM corresponded to those reported by DLS. This shape and particle size distribution have been reported, when obtaining melanin nanoparticles by *bottom-up* methods, using the conventional tyrosine oxide-reduction process [26]. In contrast, for the RMNP-HP obtained by the top-down approach with the HP method, the nanoparticles presented a morphology that described structures with an equivalent particle size similar to those measured by DLS, but with an irregular shape (Figure 3C: RMNP-HP); this characteristic may be explained as being due to the size-reduction mechanism that materials undergo with the high-pressure homogenization technique, which occurs by impact, thus provoking interparticle interactions and shocks against the walls during confinement within the impact chamber in the homogenizer [50,51].

**Figure 4:**
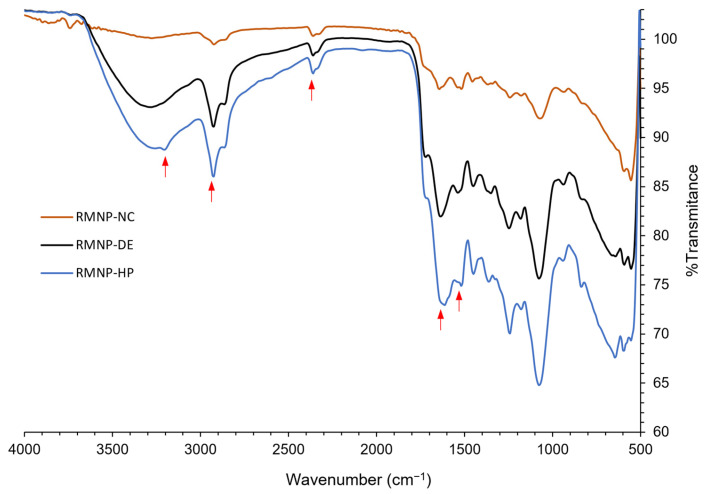
IR spectra of the recombinant melanin nanoparticles (RMNPs) prepared by the following methods: NC = nanocrystallization, DE = double emulsion–solvent evaporation, and HP = high-pressure homogenization. The red arrows show the absorption peaks related to the known functional groups of melanin.

IR spectra were recorded for the recombinant melanin nanoparticles (RMNPs) obtained by means of optimal manufacturing conditions, for the *bottom-up* and *top-down* methods. The spectra evidenced the characteristic peaks that correspond to the functional groups previously mentioned for preformed recombinant melanin (PRM), which were also present in the RMNP spectra. As observed in Figure 4, in comparison to the IR spectrum of PRM, at 3200 cm^−1^ the peak of the -NH and -OH groups was observed; at 2920 cm^−1^, the peak that corresponds to the -CH bond; at 1680 cm^−1^, there appeared a strong signal attributed to the -C=O bond of -COOH groups; at 1520 cm^−1^ the vibration of the aromatic group bonds C=C was observed again, and finally, the peak related to the protonated amine of the DHICA at 2385 cm^−1^ was detected. New peaks that could evidence the formation of new bonds were not appreciable, while small displacements in some peaks could be explained by the reordered crystalline state that melanin acquires when nanoparticles are formed [52]. In addition, these results show that the chemical integrity of the PRM was maintained after applying the different methods to produce the RMNPs.

**Figure 5:**
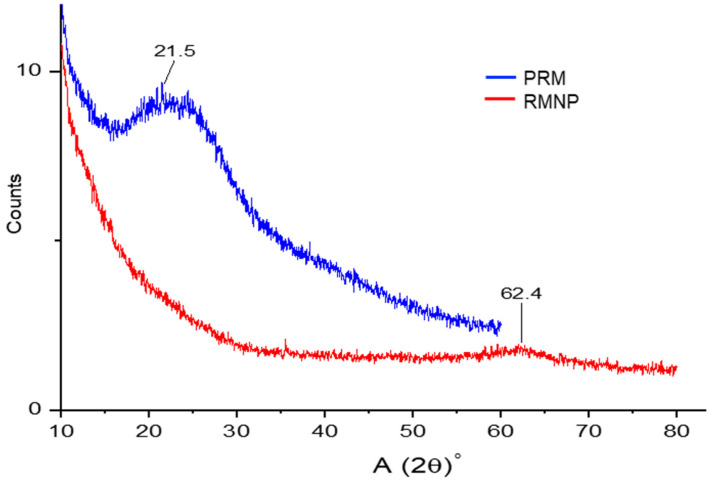
PXRD diffractograms of recombinant melanin (PRM) as raw material and the recombinant melanin nanoparticles (RMNPs).

In addition, PXRD analysis was performed on the melanin samples, preformed recombinant melanin (PRM) as raw material, and recombinant melanin nanoparticles (RMNPs). The broad peaks observed in the PXRD analysis depicted an amorphous state for PRM and treated melanin (RMNP). As observed in Figure 5, a broad band presented a 2θ value at 21.5° in PRM that was attributed to an amorphous solid state. This state for melanin has been previously reported, even for melanin from other sources, including from another biological origin [53,54]. This result also confirms that the structure and common amorphous solid state of PRM was preserved due to the stacking of molecular planes in the melanin structure that contains a range of distinct macromolecules or oligomeric material (chemical disorder model) [55]. Similarly, the RMNPs exhibited non-crystalline signals, only a broad band with 2θ value at 62.4°, suggesting a lack of sheet stacking and no order in aggregation. However, a low formation of protomolecules that aggregate by hydrogen bonding, or aggregates generated by a smaller number of protomolecules, could be attributed to the nanoparticle preparation method, the dissolution process, or both [55].

**Figure 6:**
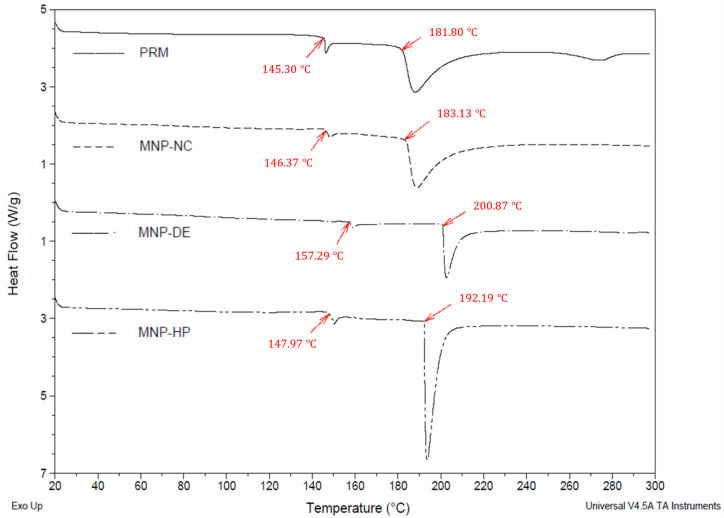
Thermograms by DSC for preformed recombinant melanin (PRM) and the recombinant melanin nanoparticles (RMNPs) obtained by the following methods: NC = nanocrystallization; DE = double emulsion–solvent evaporation, and HP = high-pressure homogenization.

The thermal behavior of the PRM and of the RMNPs is shown in the thermograms obtained by DSC, which are presented in Figure 6. For the PRM, a raw material without treatment, an endothermic peak onset temperature at 181.80 °C was recognized as its melting point or degradation peak [54], which was apparent without any significant displacement in the RMNP-NC (183.13 °C). However, a displacement of approximately 10 °C was observed in the melting point for RMNP-HP (onset temperature at 192.19 °C), which could be explained by an increase in the arrangement of melanin in the nanostructure during the manufacturing process; this new order in the solid state may be caused by the high energy added to the material when the size reduction is occurring due to pressure and impact forces. On the other hand, RMNP-DE demonstrated a displacement of 20 °C (onset temperature at 202.58 °C) in comparison to PRM, which could be explained by the order state that melanin molecules acquire during the crystallization process; this was because a slow evaporation rate was needed for the organic phase in comparison to the other two methods applied [56]. As previously mentioned, these movements at the melting point could be associated with the different degree of ordering of the melanin polymer chains due to the processes applied. 

Additionally, an endothermic event at 145.30 °C was observed in the thermograms (Figure 6), which corresponded to a first-order solid–solid transition in the solid state of the material (PRM). The latter was also observed in a similar manner for the RMNP-NC (146.37 °C), but was displaced in RMNP-DE (157.29 °C) and RMNP-HP (147.97 °C) due to changes in the arrangement of the melanin units inside the nanoparticle. The difference in degrees between the melting point and the vertex of the fusion endotherm was less than 2 °C for RMNP-DE and RMNP-HP, indicative of a higher purity for the recovered nanomaterial due to a lower amount of residue from the other materials that were added during the production process; the type of solvents used and the washing favored this condition. However, for PRM and RMNP-NC, there was a difference of 6–7 °C, which could be associated with an impurity content of at least 0.5–1% [57].

Considering the desired size of the particle, its distribution, and variability, the more reproducible manufacturing method, the lower prediction error in the modeling behavior, a solid, spherical, and compact shape for the RMNPs, the best purity after the recovery process, a Z-potential greater than ± 30 mV to favor physical stability in suspension with no aggregation phenomena, and a good chemical stability in the solid state, we decided to use the RMNP-DE for further studies. In this study, the first aim in the application of the RMNPs was related to treating or solving skin complications, such as tissue regeneration, diseases involving pigment deficiencies, drug delivery, or sun protection. Since the RMNP-DE have an expected use in health and cosmetics, biocompatibility assays with this nanomaterial were carried out using two non-carcinogenic-derived skin cell lines.

### 3.5. Biocompatibility Studies

**Figure 7:**
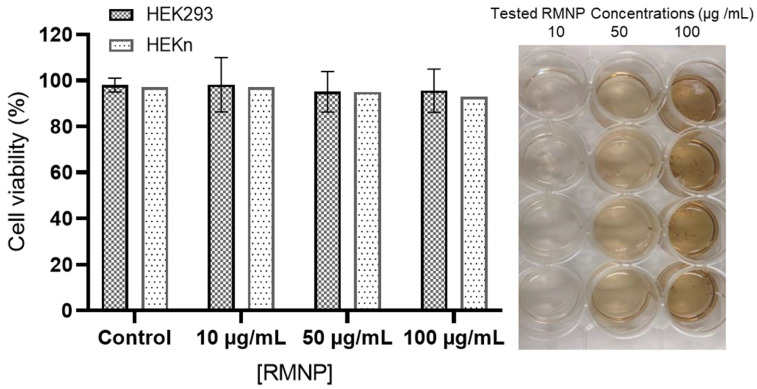
Percentage of cell viability of HEK293 and HEKn cultures using different concentrations of RMNP-DE. Three different concentrations were tested (10, 50 and 100 µg/mL). Each of the bars represents the mean of n = 8.

Once the physical and chemical characterization of the different RMNPs had been carried out, those obtained by the DE method were used for the biocompatibility analyses. These analyses were performed using two non-carcinogenic established cell lines (HEK293 and HEKn); both cell lines were selected by considering the potential applications of the RMNPs, for biomedical and cosmetic devices. Figure 7 depicts the effect of RMNPs of biotechnological origin on cell viability measured by two different techniques at 72 h after exposure to the nanoparticles. As is already known, the Trypan Blue technique is utilized to determine the % of cell viability in HEKn cells considering membrane integrity, and the MTS technique is useful for measuring mitochondrial activity in HEK293, which is directly correlated with the % of cell viability. The tested RMNP concentrations were 10, 50, and 100 µg/mL. As observed, in both cell lines and using both methodologies, the average viability did not decrease from 95%, implying that RMNPs are not toxic and allow cell-culture development for in vitro studies. In contrast, in the findings reported by Blinova et al. [5], a marked inhibition in cell proliferation was exhibited at concentrations of 50 and 100 µg/mL of melanin from black yeast fungi.

Considering that the RMNPs were steam-heat sterilized for the development of these experiments, it is important to note that the sterilization process was also effective, and no contamination was present in the cultures. This is an important fact as the cell culture media was formulated without antibiotics.

## 4. Conclusions

Melanin nanoparticles from recombinant preformed melanin were obtained applying *top-down* and *bottom-up* nanotechnology approaches, adapting three conventional manufacturing methods and starting from an insoluble raw material to obtain the nanostructures. The identity and apparent solubility of this melanin from a biotechnological origin were also characterized. Moreover, by applying experimental designs, statistical techniques, and response surface methodology, it was possible to optimize each of the three methods used for the preparation of RMNPs, in terms of the control of the particle size: nanocrystallization (NC), double emulsion–solvent evaporation (DE) and high-pressure homogenization (HP). The obtained RMNPs in all of the employed manufacturing methods presented properties that demonstrated their physical and chemical stability. In particular, the DE method evidenced the production of RMNPs in a reproducible way, generating nanostructures with a spherical and consolidated solid shape, with an average particle size of 250 ± 50 nm and an ideal Z-potential (>30 mV) to assure good physical stability and dispersion in aqueous suspension, without the formation of aggregates. The nanoparticles generated in this study showed the technological characteristics necessary for application in different areas, including medicine and cosmetics, in that they also proved to be sterilizable and biocompatible in HEK293 and HEKn cell lines. This nanomaterial could be utilized to formulate products with applications in radiation receptors in cancer therapy, as sunscreen, or in makeup to solve aesthetic skin problems (such as burned skin and vitiligo), and they may potentially be employed as contrast agents for the diagnosis of diseases or as drug delivery systems.

## Figures and Tables

**Table 1:** Experimental designs to produce and optimize RMNPs, which were used for each manufacturing process.

	*Bottom-Up* Approach										*Top-Down* Approach			
	Nanocrystallization (NC)					Double Emulsion–Solvent Evaporation (DE)					High-Pressure Homogenization (HP)			
	Factor-Levels			Response Variables		Factor-Levels			Response Variables		Factor-Levels		Response Variables	
Run	X_1_	X_2_	X_3_	Y_1_	Y_2_	X_1_	X_2_	X_3_	Y_1_	Y_2_	X_1_	X_2_	Y_1_	Y_2_
1	30	0.5	1	590.9	−2.3	10	6.66:3.33	5	167.5	−31.2	18,000	8	317.2	−38.6
2	10	0.5	1	958.6	−33.3	10	10:00	10	874.3	−25.5	23,000	10	340.6	−41.6
3	30	0.5	5	358.9	−7.6	20	13.33:6.66	5	513.8	−31.0	12,000	5	505.1	−35.3
4	10	1	5	270.4	−12.4	20	20:00	5	232.9	−39.8	23,000	5	408.9	−37.8
5	10	0.5	5	160.0	−19.1	20	13.33:6.66	10	839.7	−27.8	12,000	10	402.8	−38.5
6	30	1	5	687.0	−1.0	20	20:00	10	455.8	−18.9	X_1_: Pressure (psi) X_2_: Number of cycles			
7	30	1	1	568.9	−30.1	10	10:00	5	496.8	−23.3				
8	10	1	1	549.7	−2.8	10	6.66:3.33	10	484.1	−23.7				
	X_1_: Volume of HCl solution (mL) X_2_: HCl concentration (N) X_3_: PVAL concentration (%*w*/*v*)					X_1_: Volume of O phase (mix of DCM-ACE) (mL) X_2_: DCM:ACE ratio (mL) X_3_: Homogenization time (min)								
	Y_1_: Particle size of RMNPs (nm) Y_2_: Z-potential (mV)													

**Table 2:** RMNPs prepared under optimized conditions: model verification; n = 3 batches per process. Optimization targets: Y_1_ = 250 nm and Y_2_ = maximized response.

Method	Factors	Estimated Levels	$\;\;\;{\hat{Y}}_{1}\;(\mathrm{nm})$	D	Y_1_ (nm)	PEE (%)	Y_2_ (mV)	
NC	X_1_: Volume of HCl solution	10 mL	250.2	0.86	245.9 ± 31.5	1.72	−20.2 ± 1.56	
	X_2_: HCl concentration	0.56 N						
	X_3_: PVAL concentration	4.7 % *w*/*v*						
DE	X_1_: Volume of organic phase	19.7 mL	252.7	0.89	253.1 ± 30.6	0.16	−39.2 ± 0.56	
	X_2_: DCM:ACE ratio	19.7:0 ratio (mL)						
	X_3_: Homogenization time	5 min						
HP	X_1_: Applied pressure	18,000 psi	~321.17	0.99	302.2 ± 69.9	5.91	−38.6 ± 2.25	
	X_2_: Number of cycles	10 cycles						
NC: Nanocrystallization DE: Double emulsion–solvent evaporation HP: High-pressure homogenization (HP)			${\;\hat{Y}}_{1}:$ Predicted particle size Y_1_: Observed particle size Y_2_: Z-Potential D: Desirability PEE: Percentage of estimation error					

**Table 3:** Apparent solubility of PRM; practically insoluble: < 0.1 mg/mL, and slightly soluble: between 1 and 10 mg/mL.

Test Solvent	Apparent Solubility
Distilled water (pH~6)	Practically insoluble
NaOH 1N (pH > 12)	Slightly soluble (1.10 mg/mL)
NH_4_OH 1N (pH > 12)	Slightly soluble (0.98 mg/mL)
HCl 1N (pH 1.2)	Practically insoluble
Ethanol	Practically insoluble
Isopropyl alcohol	Practically insoluble
n-Butanol	Practically insoluble
Acetone	Practically insoluble
Glyceryl triacetate	Practically insoluble
Octanol	Practically insoluble
Ethyl acetate	Practically insoluble
Dichloromethane	Practically insoluble
Benzene	Practically insoluble
Hexane	Practically insoluble

## Data Availability

The data presented in this study are available on request from the corresponding author.

## Supplementary Materials

The following supporting information can be downloaded at: https://www.mdpi.com/article/10.3390/polym15102381/s1, Figure S1: Preformed recombinant melanin (PRM); (A) granular raw material, (B) pulverized raw material; Figure S2: Pareto charts of main effects on particle size for the three manufacturing methods employed to produce RMNPs; (A) nanocrystallization (NC), (B) double emulsion–solvent evaporation (DE), and (C) high-pressure homogenization (HP).
