# Supplementary material for: Melanin Nanoparticles Obtained from Preformed Recombinant Melanin by Bottom-Up and Top-Down Approaches
Source: Polymers (Basel). 2023 May 19;15(10):2381. doi: 10.3390/polym15102381 (PMC10224555; doi:10.3390/polym15102381)
Supplement: Supplementary file 1 [file polymers-15-02381-s001.zip › polymers-2320457-supplementary.pdf]

Supplementary Materials

Melanin Nanoparticles Obtained from Preformed Recombinant Melanin by *Bottom-Up* and *Top-Down* Approaches

Sergio Alcalá-Alcalá [1], José Eduardo Casarrubias-Anacleto [1], Maximiliano Mondragón-Guillén [2], Carlos Alberto Tavira-Montalvan [2], Marcos Bonilla-Hernández [1], Diana Lizbeth Gómez-Galicia [3], Guillermo Gosset [4] and Angélica Meneses-Acosta [2,*]

[1] Laboratorio de Investigación en Tecnología Farmacéutica, Facultad de Farmacia, Universidad Autónoma del Estado de Morelos, Cuernavaca, Morelos,62209, Mexico; sergio.alcala@uaem.mx ((S.A.-A.); jose.casarrubiasana@uaem.edu.mx (J.E.C.-A.); marcos.bonillahz@gmail.com (M.B.-H.)

[2] Laboratorio de Biotecnología Farmacéutica, Facultad de Farmacia, Universidad Autónoma del Estado de Morelos, Cuernavaca, Morelos, 62209, Mexico; maximiliano.mondragong@uaem.edu.mx (M.M.-G.); carlos.taviramon@uaem.edu.mx (C.A.T.-M.)

[3] Farmacia Hospitalaria, Facultad de Farmacia, Universidad Autónoma del Estado de Morelos, Cuernavaca, Morelos, 62209, Mexico; diana.gomezg@uaem.edu.mx

[4] Departamento de Ingeniería Celular y Biocatálisis, Instituto de Biotecnología, Universidad Nacional Autónoma de México, Cuernavaca, Morelos, 62209, Mexico; guillermo.gosset@ibt.unam.mx

* Correspondence: angelica_meneses@uaem.mx; Tel.: +52-7773297000 (ext. 3366)

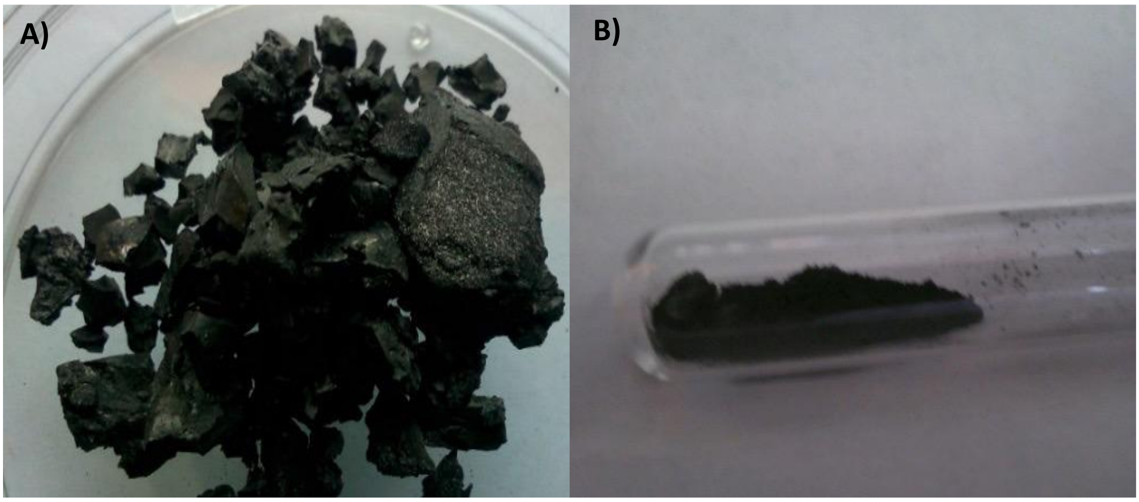

Figure S1. Preformed recombinant melanin (PRM); A) granular raw material, B) pulverized raw material.

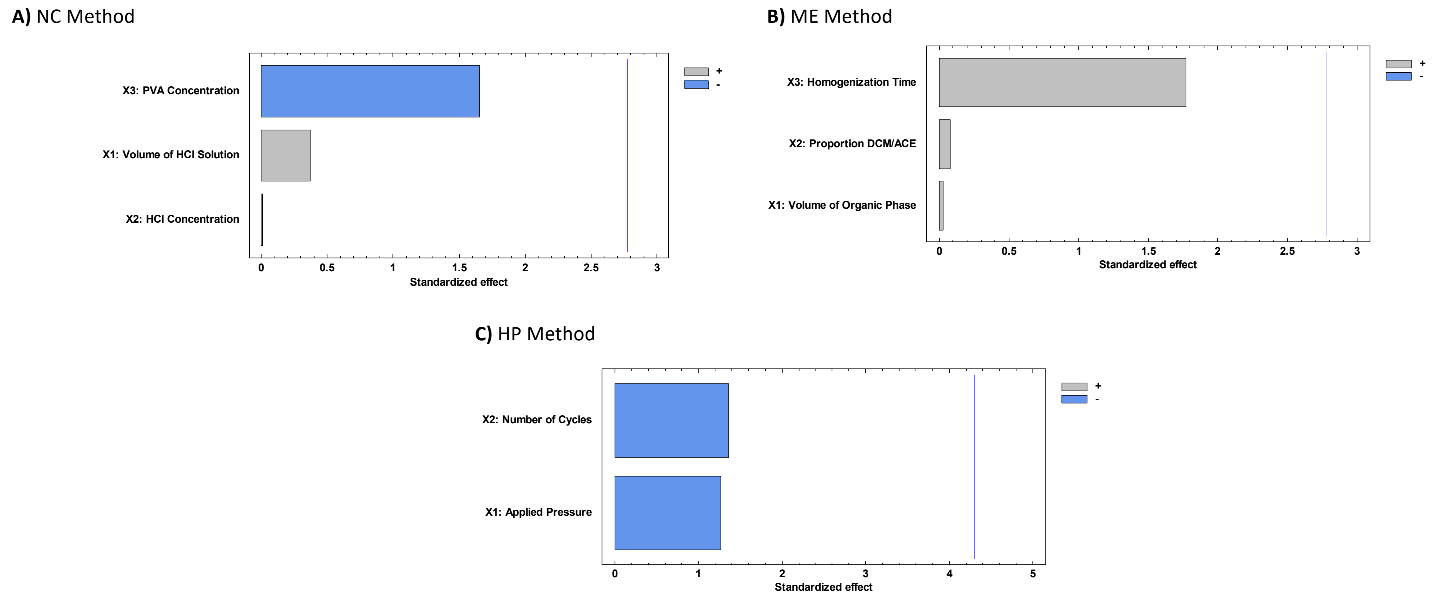

Figure S2. Pareto charts of main effects on particle size for the three manufacturing methods employed to produce RMNP; A) Nanocrystallization (NC), B) Double Emulsion - Solvent Evaporation (DE), C) High Pressure Homogenization (HP).
